# Using NLP in openEHR archetypes retrieval to promote interoperability: a feasibility study in China

**DOI:** 10.1186/s12911-021-01554-2

**Published:** 2021-06-26

**Authors:** Bo Sun, Fei Zhang, Jing Li, Yicheng Yang, Xiaolin Diao, Wei Zhao, Ting Shu

**Affiliations:** 1 Department of Information Center, Fuwai Hospital, National Center for Cardiovascular Diseases, Chinese Academy of Medical Sciences and Peking Union Medical College, No. 167 North Lishi Road, Xicheng District, Beijing, 100037 China; 2 Beijing Advanced Innovation Center for Biomedical Engineering, School of Biological Science and Medical Engineering, Beihang University, 37 Xueyuan Road, Haidian District, Beijing, 100191 China; 3 Fuwai Hospital, National Center for Cardiovascular Diseases, Chinese Academy of Medical Sciences and Peking Union Medical College, No. 167 North Lishi Road, Xicheng District, Beijing, 100191 China; 4 National Institute of Hospital Administration, National Health Commission, Building 3, yard 6, Shouti South Road, Haidian, Beijing, 100044 China

**Keywords:** OpenEHR, Nature language processing, Information retrieval, Interoperability

## Abstract

**Background:**

With the development and application of medical information system, semantic interoperability is essential for accurate and advanced health-related computing and electronic health record (EHR) information sharing. The openEHR approach can improve semantic interoperability. One key improvement of openEHR is that it allows for the use of existing archetypes. The crucial problem is how to improve the precision and resolve ambiguity in the archetype retrieval.

**Method:**

Based on the query expansion technology and Word2Vec model in Nature Language Processing (NLP), we propose to find synonyms as substitutes for original search terms in archetype retrieval. Test sets in different medical professional level are used to verify the feasibility.

**Result:**

Applying the approach to each original search term (n = 120) in test sets, a total of 69,348 substitutes were constructed. Precision at 5 (P@5) was improved by 0.767, on average. For the best result, the P@5 was up to 0.975.

**Conclusions:**

We introduce a novel approach that using NLP technology and corpus to find synonyms as substitutes for original search terms. Compared to simply mapping the element contained in openEHR to an external dictionary, this approach could greatly improve precision and resolve ambiguity in retrieval tasks. This is helpful to promote the application of openEHR and advance EHR information sharing.

## Background

With the development of big data processing technology, the effective use of medical data inevitably became a trend [1]. The improvement of the quality of medical services, the reduction of medical service costs, and even the progress and development of medicine have become increasingly dependent on the effective use of medical data [2–4]. More recently, the Electronic Health Record (EHR) has been defined as a viable source of data for regulatory decision-making [5]. However, bias can occur along the various steps of the data chain, and can lead to unusable data or invalid analysis results [6]. The lack of semantic interoperability is cited as a primary reason for inefficiencies within the healthcare system in the United States, contributing to billions of wasted dollars annually [7, 8]. Thus, semantic interoperability [9] is essential for accurate and advanced health-related computing and EHR sharing [10, 11]. Medical information models are used to improve semantic interoperability [12, 13]. Currently, main-stream medical information models about semantic interoperability include: HL7-V3 [14], FHIR [15], ISO13606 [16–18], openEHR [19], and more. In this regard, openEHR is of particular interest because a large community of developers and many open-source tools are available [20]. OpenEHR has already been implemented in several countries (e.g. the United Kingdom, Australia) and is attractive to developing countries [21–23]. The Medical Software Branch and the Smart and Mobile Medical Branch of the China Association for Medical Devices Industry jointly established openEHR China in March 2016. In 2018, Min et al. built Chinese archetypes that were uploaded to the Healthcare Modelling Collaboration (HMC) [24]. HMC manages archetypes and facilitates the reuse of the same archetypes in China.

The openEHR approach can improve semantic interoperability [25, 26]. One key improvement in openEHR compared to other systems is that, as the name ‘open’ implies, it allows for the use of both existing and newly created archetypes [27]. Archetype plays an important role in the openEHR approach, as it supports not only semantics but also scalability and interoperability [25]. The crucial problem is finding the relevant archetypes from open repositories [28]. It is difficult to achieve this goal. These concept names of archetypes are described by professional medical terms [25]; however, some users, including patients [29], may use layperson wording for terms when searching target archetypes [30]. Similar to searching in PubMed, the most common reason for retrieval error is a lack of synonymous terms [32].

In recent years, the importance of synonym-learning which may help alleviate the lack of synonyms is well recognised in the NLP research community, especially in the biomedical [30] and clinical domains [31]. The most important part of synonym-learning is semantic extraction. With the development of NLP technology such as multi-label text categorization [32], text generation [33] and so on, there are many researches about semantic extraction. Yang et al. [34] combined reinforcement learning, generative adversarial networks, and recurrent neural networks to build a termed category sentence generative adversarial network (CS-GAN), which can help to generate synonymous sentences so that enlarge the original dataset. Younas et al. [35] manipulated the textual semantic of functional requirements to identify the non-functional requirements in software development and used the similarity distance between the popular indicator keywords and requirement statements to identify the type of non-functional requirement. In their study, the semantic similarity is calculated based on co-occurrence of patterns in large human knowledge repositories of Wikipedia. Liu et al. [36] proposed a novel end-to-end multi-level semantic representation enhancement network (MLSREN) which can enhance the semantic representation of entities from word, phrase, and context level. With the semantic representation of words, we can calculate the similarity between them, and find synonymous terms which can be used to expand the user’s search terms and realize query expansion.

Crimp et al. [37] used the semantic dictionary WordNet to expand the query, then refined the candidate expansions by discriminating relevancy and excluding spurious expansion terms which help reduce query drift and increase query performance. ItiChaturvedi et al. proposed a Variable-order Belief Network (VBN) framework, which is good at modeling word dependencies in text, can be used for semantic representation of words [38]. Similarly, Huang et al. [39] used the deep belief network (DBN) model to capture the meaningful terms for effective query expansion in the code searching task. The model both extracts relevant terms to expand a query and excludes irrelevant terms from the query and outperforms several query expansion algorithms for code search. Yusuf et al. [40] enhanced the query expansion method based on unigram model and word embedding using Glove, which can capture the semantic similarity. The results show that Glove’s [41] model for word embedding can significantly improve query expansion methods using Arberry dataset. Another famous Word embedding model is Word2Vec, developed by Mikolov et al. [41] which represents words with a continuous vector obtained from a neural network model trained on a huge text corpus. Since the word vector contains the semantic information of the word, the similarity between the two words is accurately reflected by the distance of the word vector. At present, Word2Vec is widely used in the calculation of word similarity.

Aim of this study is resolve mistakes related to ambiguity and promote semantic interoperability. Therefore, we proposed and assessed an approach using NLP technology and corpus to expand search terms by finding synonyms as alternative terms. We also sought to verify the process by testing examples taken from Chinese archetypes and corpus.

## Methods

### Approach description

**Fig. 1:**
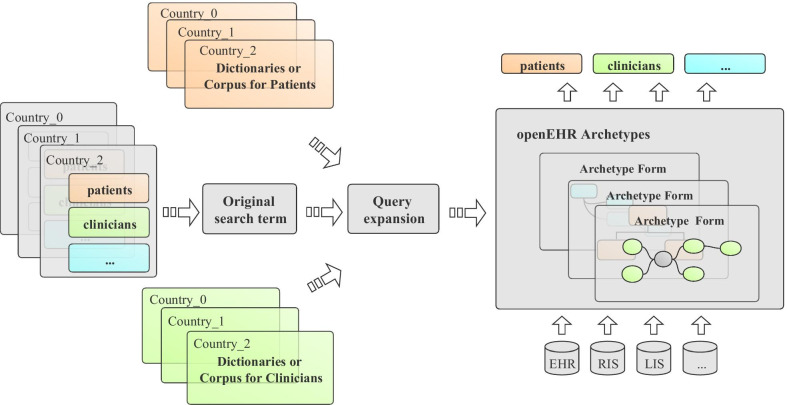
Approach description of this study. EHR: electronic health records; RIS: Radioiogy information system; LIS: Laboratory Information Management System

For an original search term, we use the query expansion technology to find its synonyms as a substitute to search the target archetype in openEHR (Fig. 1). By using this in archetype retrieval, we can choose dictionaries or corpus in different fields to expand the search terms entered by people who with different backgrounds. This ability is essential for improving openEHR’s interactivity, retrieval precision and application in different regions or countries. Such as expanding radiologist search terms by RadLex, expanding search terms of clinicians by Unified Medical Language System (UMLS) Metathesaurus, Systematized Nomenclature of Medicine Clinical Terms (SNOMED‐CT) and expanding search terms used by patients (people who without medical knowledge) by Word-Net, Wikipedia, and so forth. Using the dictionaries and corpora of different countries also allows us to expand the search terms entered in different countries.

**Fig. 2:**
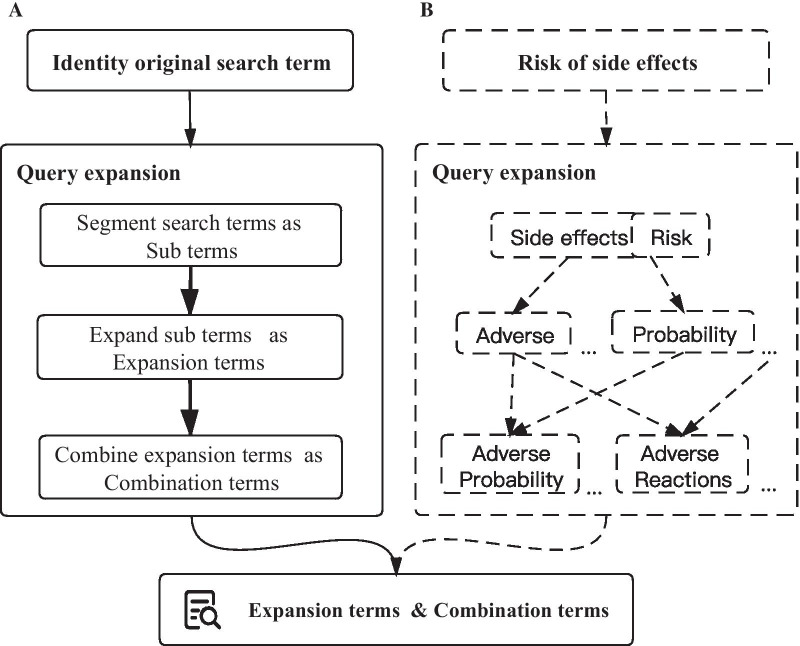
Process of query expansion. **a** Three key steps of query expansion: term segmentation, term expansion and term combination; **b** Example of query expansion: risk of side effects

There are three key steps of query expansion: term segmentation, term expansion and term combination (Fig. 2). Term segmentation is dividing the original search term into a sub term. Term expansion is the use of NLP technology and materials to further provide synonyms for the sub terms. Term combination is to combine expansion terms to form combination terms. Finally, expansion terms and combination terms are used as substitutes of the original search term to search archetypes in openEHR.

To prove the feasibility of the approach, we selected the Chinese openEHR archetypes as data sources, built test sets and chose the Chinese Wikipedia data as the expanded corpus.

### Data sources

The Chinese archetypes are stored by HMC. Having only 64 archetypes when it was created in 2018, but now it includes 410 archetypes. Among these, 16 concept names of archetypes are described only in English, so we used the other 394 archetypes as the data source.

### Test sets

**Fig. 3:**
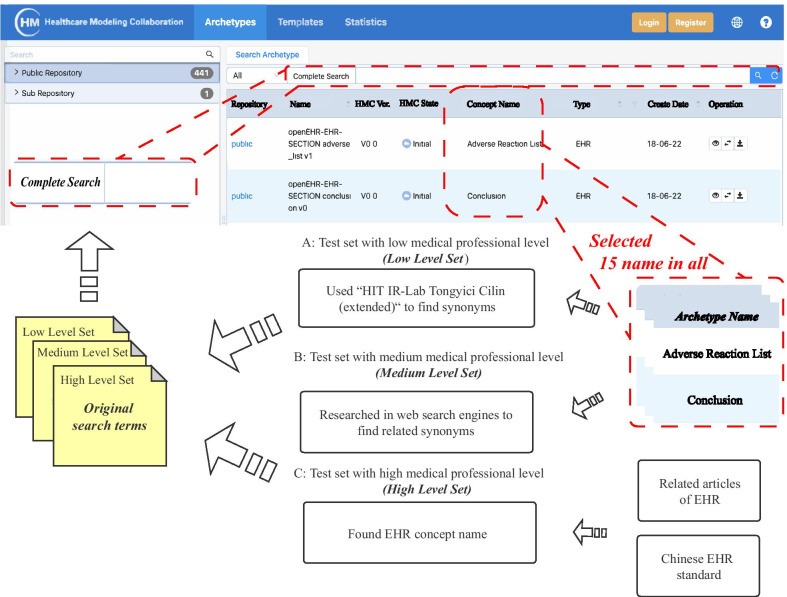
Process for constructing test sets in different medical professional level: low, medium and high

In order to simulate the real input search term to the greatest extent, we constructed test sets with different medical professional levels: low, medium and high. The construction follows these principles: first, search terms should be relevant to the Chinese EHR; second, search terms should reuse some clinical content, such as medical events prediction, clinical research and disease research. We defined the content contained in the test sets as original search terms, a total of 45 original search terms was constructed (Low Level Set:15, Medium Level Set:15, High Level Set:15). The directly searching of target archetypes in HMC with original search term was definded as baseline mode (Fig. 3).

#### Test set with low medical professional level (Low Level Set)

We divided all archetypes stored in the data source (published in HMC) into three parts: basic patient information, medication and clinical examination. For each part, we randomly selected five archetypes, a total of 15 target archetypes. We used ‘HIT IR-Lab Tongyici Cilin extended version (TC-E)’ [42] to find related synonyms of target archetypes’ Chinese names as original search terms. TC-E is an authoritative semantic dictionary in the common languages field of Chinese [43]. For example, ‘health summary’ is the Chinese name of archetype openEHR-EHR-COMPOSITION-health-summary.v1. Searching in TC-E, we found that ‘health abstract’ is a synonym of it. Hence, ‘health abstract’ was used as original search terms for archetype openEHR-EHR-COMPOSITION. health-summary.v1. In the end, a total of 40 terms was generated (Table 1). Since TC-E is a collection of common Chinese languages, we assumed this test set’s medical professional level is ‘low’.

**Table 1:** Description of test sets

Test sets	Target archetype		Original search terms	
	Examples	Number, n	Examples	Number, n
Low level set	Skeletal age, medicine management, etc	15	Bone age, drug management, etc	40
Medium level set	Fetal heartrate, care program, etc	15	Fetal heartbeat, care plan, etc	40
High level set	NA	NA	Blood transfusion label, admission diagnosis, etc	40

#### Test set with medium medical professional level (Medium Level Set)

 For better contrast, we chose the target archetypes in Low Level Set as the target archetypes. We searched these archetypes’ Chinese names in online search engines (Bing, Google, etc.) to find related terms as original search terms. For example, we searched ‘fetal heartrate’, the content name of the archetype openEHREHROBSERVATION.fetal heart.v1 in Bing and got medical science articles contained ‘fetal heartbeat’ [10]. Hence, we use ‘fetal heartbeat’ as original search terms for the archetype openEHREHROBSERVATION.fetal heart.v1. Repeating the above operations, a total of 40 terms were found (Table 1). Because the test set is composed of related terms in popular science articles and medical Q&A websites data, we assumed its medical professional level was ‘medium’.

### Test set with high medical professional level (High Level Set).

By searching ‘ehr’ or ‘emr’ in PubMed, we screened the literature with relevant data published over a period of 20 years and found the EHR data provided by relevant studies. Combining with the EHR data mentioned in the literature and the ‘Basic Data Set of Electronic Health Record’ issued by the China Health Construction Commission, we chose some content names of the EHR as original search terms to construct the test set (Table 1). Because the test set is composed of element names in EHR, we assume its medical professional was ‘high’.

### NLP and query expansion

Query expansion (popular in text retrieval community) is a technique used to improve search precision. The basic idea is using results from an initial query to reformulate the query and then get more precise results. Data sources used in query expansion methods rely on external lexical-semantic resources, typically dictionaries or other similar knowledge representation resources.

### Term segmentation

Word segmentation is an important preprocessing step in several NLP systems [44] such as machine translation [45], information retrieval [46], etc. Existing word segmentation algorithms can be roughly divided into four categories [47]: manual rules-based, dictionaries-based, traditional machine learning-based and deep learning-based. Considering the complexity of the system and segmentation precision, in this study, we chose the word-segmentation algorithm based on traditional machine learning to accomplish the segmentation task.

### Term expansion

**Fig. 4:**
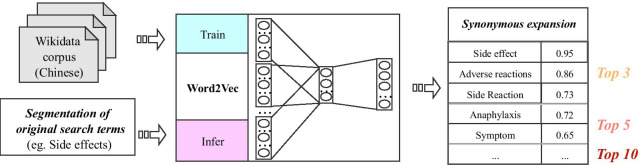
The illustration of synonym-expansion based on the Word2Vec. At train stage, Wikidata in Chinese is used to learn the representation vector of words, and then use the cosine distance to calculate the similarity between user’s term with all terms in vocabulary, after ranking, return top 10 terms as the expansion of user’s term

Synonym expansion is the next step for resolving ambiguity and improving the precision of the archetype retrieval after segmenting the original terms. In the medical field, there is currently no high-quality lexicon of Chinese synonyms. Word embedding technology that has emerged in recent years is expected to solve this problem, which can express words as continuous vectors—the distance between two words in space can indicate the semantic connection or similarity of them. Word2Vec [48], uses context information for training, maps words into a high-dimensional space and uses the distance in the high-dimensional space as the basis for calculating the semantic similarity between two words. Therefore, at the algorithm level, retrieval is based on more ‘semantic distance’ than ‘rule matching’. Based on this idea, we aimed to implement synonym-expansion based on the Word2Vec trained by the Wikidata-corpus (Fig. 4).

### Assessment

HMC provide two search methods: ‘Normal search’ and ‘Completes search’. We choosed ‘Completes search’ as search method since it could search more content.

**Fig. 5:**
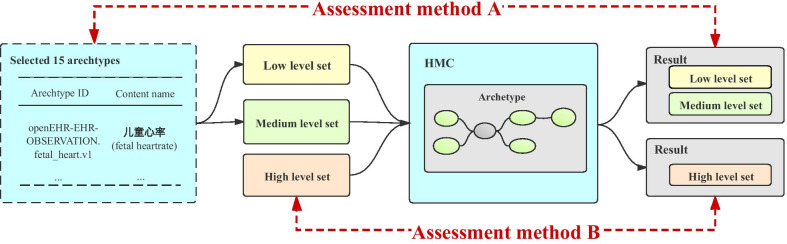
Two methods for assessingtest sets. Assessment method A: for Low and Medium Level Set, compare the result with ID in the original archetypes to determine; Assessment method B: for High Level Set, recruit two volunteers with clinical backgrounds to examin the results combined with the original EMR data information to determine

For Low and Medium Level Set, since the original search terms are derived from the 15 target archetypes that have been selected in HMC, we could compare the search result return by HMC with target archetypes to determine which is true. Meanwhile, for each original search term, there were many related expansion and combination terms (Tabel 4), and multiple search results. In this condition, we use Word2vec to calculate the semantic similarity of each results’ archetype name and original search term. Sorting these results in descending order, according to the relevance (Fig. 5, Assessment method A).

For High Level Set, due to these original search terms are derived from EMR data, we did not know which search result is true. In this condition, two volunteers with clinical backgrounds were recruited. They examined the results combined with the original EMR data. A total of 5 part needed to be manually assessment, especially, results of original search terms, results of expansion terms (Top3 and 5) and results of combination terms (Top3 and 5). Two volunteers assessed these results, back to back. For the inconsistent assessments, the two sides discussed and got the final result (Fig. 5, Assessment method B).

Precision at K (P@K), average precison (AP) and mean average precision (MAP) are used as evaluation metrics. The precision at K (P@K) is calculated as follows:

1 $$\begin{array}{*{20}c} P@K = ~\frac{{\mathop \sum \nolimits_{{i = 0}}^{N} \delta \left( {i,K} \right)}}{N} \\ \end{array}$$

$\delta \left( {i,K} \right)$ is an indicator function which equals 1, if there is an acceptable result among the top K returned by HMC or equals 0. $N$ is the total number of test set (low level test: 40, medium level test: 40, high level test: 40). In this study, K is 3 and 5 respectively (P@3, P@5).

The average precision (AP) is a measure that combines recall and precision for ranked retrieval archetypes. For one test set, AP is the mean of the P@r after top $~r$ relevant archetypes are retrievel for each search term (origianl search terms or expansion terms or combination terms), $r \in \left\{ {3,5} \right\}$.

2 $$\begin{array}{*{20}c} AP = ~\frac{1}{R}\mathop \sum \limits_{r} P@r \\ \end{array}$$

The mean average precision (MAP) is the arithmetic mean of the AP values for a retrieval system over a set of $n$ test sets. $n \in \left\{ {low\;~level\;~set,~medium\;~level~\;set,high~\;level\;~set} \right\}$. It can be expressed as follows:

3 $$\begin{array}{*{20}c} MAP = \frac{1}{N}\mathop \sum \limits_{n} AP_{n} \\ \end{array}$$

## Results

### Overview of expansion results

For each original search terms in the test sets, in the process of synonym expansion, the first 3, 5, and 10 terms of similar results were used as expansion terms and were combined into combination terms, which are recorded as Top 3, Top 5, and Top 10. Take the original search terms ‘Risk of side effects’ as an example: Table 2 shows the construction process of its expansion terms; Table 3 shows the construction process of its combination terms. For all 120 original search terms, a total of 4729 expansion terms and 64,619 combination terms were constructed (Table 4).

**Table 2:** Process for constructing expansion terms (for example: risk of side effects)

Original search term	Segmentation terms	Expansion terms		
		Top 3 (sum: 6)	Top 5 (sum: 10)	Top 10 (sum: 20)
Risk of side effects	Side effects	Side effect	Side effect	Side effect
		Adverse reactions	Adverse reactions	Adverse reactions
		…	Untoward reactions	Untoward reactions
			…	Anaphylaxis
				…
	Risk	Risk	Risk	Risk
		Possibility	Possibility	Possibility
		…	Danger	Danger
			…	Probability
				…

**Table 3:** Process for constructing combination terms (for example: risk of side effects)

Original search term	Combination terms		
	Top 3 (sum: 9)	Top 5 (sum: 25)	Top 10 (sum: 100)
Risk of side effects	Side effect risk	Side effect risk	Side effect risk
	Side effect possibility	Side effect possibility	Side effect possibility
	…	Adverse reactions risk	Adverse reactions risk
		Adverse reactions possibility	Adverse reactions possibility
		…	Untoward reactions risk
			Untoward reactions Possibility
			…

**Table 4:** Results for constructing expansion terms & combination terms

Test queries	Original search term, n	Expansion term, n			Combination term, n		
		Top 3	Top 5	Top 10	Top 3	Top 5	Top 10
Low level set	40	216	360	719	312	840	3279
Medium level set	40	267	455	891	546	2085	13,712
High level set	40	303	505	1013	1050	6300	36,486

### Evaluation of the performance

Manual assessment results of High Level Set are shown in Table 5. From the table, we can see that the two volunteers did agree on the search results of original search terms but difference on that of expansion and combination terms. After discussion again, finally result is obtained as the result of High Level Set.

**Table 5:** Manual assessment results of ‘High Level Set’

Methods		Volunteer A			Volunteer B			Finally result		
		AP	P@3	P@5	AP	P@3	P@5	AP	P@3	P@5
Original search terms	Top 3	0.150			0.150			**0.150**		
	Top 5									
	Top 10									
Expansion terms	Top 3	0.637	0.525	0.750	0.662	0.575	0.750	**0.637**	**0.525**	**0.750**
	Top 5	0.575	0.475	0.675	0.612	0.550	0.675	**0.612**	**0.550**	**0.675**
	Top 10	0.575	0.500	0.650	0.575	0.500	0.650	**0.575**	**0.500**	**0.650**
Combination terms	Top 3	0.150	0.150	0.150	0.175	0.175	0.175	**0.150**	**0.150**	**0.150**
	Top 5	0.150	0.150	0.150	0.175	0.175	0.175	**0.175**	**0.175**	**0.175**
	Top 10	0.150	0.150	0.150	0.200	0.200	0.200	**0.175**	**0.175**	**0.175**

Finally result values are emphasized in bold

**Fig. 6:**
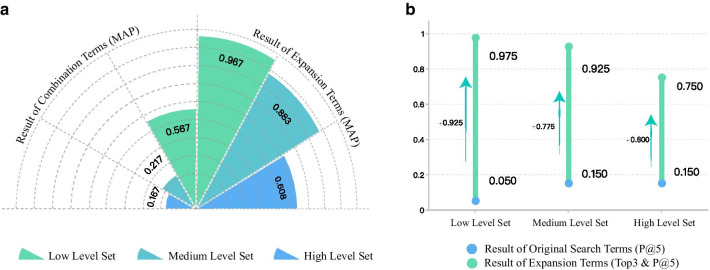
Comparison result with baseline model. **a** Comparison result between expansion term and combination terms. **b** Comparison result between our method (Expansion terms & Top 3) and baseline model

Expansion terms achieved the best mean MAP (0.819) with an MAP of 0.967, 0.883, 0.608 for each test set respectively. Combination terms achieved the mean MAP of 0.317, with an MAP of 0.567, 0.217, 0.167 for each test set respectively (Fig. 6a). For the same expansion term, the search results with different thresholds (Top3, 5, 10) are also different. According to Table 6, when the threshold is Top 3, AP = 0.963 (Low Level Set), 0.888 (Medium Level Set) and 0.637 (High Level Set). In contrast, when the threshold is Top 10, AP = 0.961 (Low Level Set), 0.875 (Medium Level Set) and 0.608 (High Level Set) Table 6.

**Table 6:** Retrieval performance comparison between expansion terms and combination terms with different similarity thresholds

Methods			AP	P@3	P@5
Low level set	Original search terms		0.050	0.050	0.050
	Expansion terms	Top3	0.963	0.950	0.975
		Top5	0.975	0.950	1.000
		Top10	0.963	0.950	0.975
		Mean	0.967	0.950	0.983
	Combination terms	Top3	0.512	0.500	0.525
		Top5	0.575	0.550	0.600
		Top10	0.6125	0.600	0.625
		Mean	0.567	0.550	0.583
Medium level set	Original search terms		0.137	0.125	0.15
	Expansion terms	Top3	0.888	0.850	0.925
		Top5	0.888	0.850	0.925
		Top10	0.875	0.850	0.900
		Mean	0.883	0.850	0.917
	Combination terms	Top3	0.200	0.200	0.200
		Top5	0.200	0.200	0.200
		Top10	0.250	0.250	0.250
		Mean	0.217	0.217	0.217
High level set	Original search terms		0.150	0.150	0.150
	Expansion terms	Top3	0.637	0.525	0.750
		Top5	0.612	0.550	0.675
		Top10	0.575	0.500	0.650
		Mean	0.608	0.525	0.692
	Combination terms	Top3	0.150	0.150	0.150
		Top5	0.175	0.175	0.175
		Top10	0.175	0.175	0.175
		Mean	0.167	0.167	0.167

Different similarity thresholds: in the process of synonym expansion, the first 3, 5, and 10 terms of similar results are used as expansion terms and then composed as combination terms

AP: average precision, P@3: precision at 3, P@5: precision at 5

In this article, K in the Formula (1) is 3 and 5 respectively, and get P@3, P@5. From Table 6, we can know that P@5 greater than P@3 on each test set. Taking Low Level Set for example: mean P@5 = 0.983 (Expansion terms) and 0.583 (Combination terms), mean P@3 = 0.949 (Expansion terms) and 0.550 (Combination terms).

By selecting Expansion terms and Top 3 as the best parameters, this method is compared with the baseline way (that is, directly use original search terms of test sets to search archetype, without any processing), which could show the superiority of this method. The results are shown in Fig. 6b. Firstly, there is no big gap among the search results of baseline method in three test set, as they are all lower than 0.2 (P@5). After using the method (Expansion Terms and Top 3), P@5 of each test set has been greatly improved. In Low Level Set, P@5 is the highest (0.975) and in contrast, High Level Set get the worst result: P@5 = 0.750. Compare to the baseline, the result of each data set is significantly improved. In Low Level Set, after using the method presented, P@5 is increased by 0.925. The same is for other two set, which are increased by 0.775 and 0.600, respectively.

## Disscusion

In order to verify this method, we used three different methods to construct test sets with different medical professional level: Low, Medium and High. P@3, P@5, AP and MAP are used as evaluation metrics. In this study, for a search term, if the target archetype appears in the first three results returned by HMC, then P@3 is 1. If the target archetype appears in the first five, then P@5 is 1. Generally speaking, for a retrieval method, P@5 will be greater than P@3[49], the same is true in our study. Baseline model, which directly uses the original search terms for retrieval, got the lowest precision on the three test sets. On the P@5 level, only 2, 6 and 6 original search terms can search the target archetype, respectively. The low precision is caused by the mismatch between the search term and the name of the openEHR archetype. With the improvement of the medical professional level of search term, the search precision has also been improved. AP is 0.050 (Low level set), 0.137 (Medium level set) and 0.150 (High level set) respectively.

The purpose of this study is to promote the interoperability in the using of openEHR. Choosing Expansion terms and Top 3 as the best parameters to compare with baseline, P@3 and P@5 are increased by 90.0% and 92.5% (Low Level Set), 72.5% and 77.5% (Medium Level Set), 37.5% and 60.0% (High Level Set). The improvement of different test set is also different. P@5 of Low Level Set is up to 0.975 and of High Level Set is up to 0.750. The difference may be due to Chinese Wikipedia data. Wikipedia is not a professional medical corpus but does contain various kinds of medical vocabulary terms. It is easy to establish a connection between professional and lay medical vocabularies for people without medical knowledge.

Regarding the test sets, Mean P@5 is improved by 0.767. It has been proven that this method has superiority and generality. This is very meaningful, for that even in the same country, the distribution of medical resources and the level of doctors are often unbalanced. Take China as an example, the level of knowledge of medical staff in China varies greatly. The primary care doctors in China have low levels of training, low job satisfaction, and high occupational stress; meanwhile, the application of information technology (IT) is fragmented [50]. This method is helpful to promote the application of openEHR and even EHR in developing countries, such as China.

Previous studies showed that using concept subnetwork structure could help us estimate semantic similarity and improve retrieval results (P@10 = 0.6) [28]. In this study, we selected P@5, which was better than P@10 as an evaluation metric as finally metrics and got P@5 = 0.883 (Expansion Tems & Top 3), on average. Our experiments also led to the following points worth noting:

### Calculating semantic relevance of queries to solve ambiguity

In the medical domain, there is one vocabulary that is used by medical professionals more frequently than others, whereas patients often use alternative and lay terms or synonyms [51]. For example, ‘medication item’ and ‘medicine item’ referred to the same terms. Yang [29] proposed a graphical retrieval method to improve archetype retrieval performance and validate the method’s feasibility. However, the method presented lacks the calculation of the semantic relevance of synonyms or homonyms for search terms. In this study, we introduced synonym-learning in NLP into openEHR innovatively to solve the retrieval errors caused by the lack of synonyms.

The essence of the NLP technology used in this article is to expand the search terms. Table 4 verifies this. Taking Low Level Set as an example, when the threshold is Top5, 360 expansion terms and 840 combination terms are expanded from 40 original search terms. Combination terms are a combination of the expansion terms. Their length is generally greater than that of expansion tems, and they also contain many grammatical error terms. Since the current search method provided by HMC is based on character matching, the longer the search term, the lower the search accuracy. From Table 6, we can see that the results of combiantion terms are lower than expansion terms on each set (Low level set: expansion terms MAP: 0.967, combination terms MAP: 0.567; Medium level set: expansion terms MAP: 0.883, combination terms MAP: 0.217; High level set: expansion terms MAP: 0.608, combination terms MAP: 0.167).

### Sorting retrieval results on semantic similarity

Table 4 illustrates that for the same original search term, as the threshold (Top 3, 5 and 10) increases, the corresponding expansion and combination term will also increase. In the example of Medium Level Set, there were a total of 40 original search terms which generated 267 expansion terms at Top 3, 455 terms at Top 5 and 891 terms at Top 10. How to integrate the returned results and present them to the searcher is a problem. This study proposes a method to solve this problem by using NLP technology, specifically: calculating the semantic relevance between name of each result and the original search term and arranging them in descending order.

### The excessive increase in the number of search terms did not improve the search precision

The core step of this method is the expansion of search terms, but the excessive increase does not lead to an increase on the search precision. According to Table 6, when the threshold of High Level Set is Top 3, AP has reached 0.637. In contrast, when the threshold is Top 10, AP is only 0.575. Although the difference between the two values is small, it is also worth noting. There are too many expansion terms and unnecessary errors are introduced. We need to choose an appropriate threshold, which in this study is Top 3.

This feasible study also has some limitations. In our experiment, we selected Wikipedia data as the expansion corpus, which led to a decline in the expansion performance of high medical professional level search terms. In the future, we can use different corpora for word expansion and compare their effects. Also, the abbreviations and acronyms lead to decreased readability and pose challenges for information retrieval [31]. In a future study, we will aim to resolve the problem of abbreviations in archetype retrieval tasks.

## Conclusion

The purpose of this study is to promote the interoperability in the using of openEHR. To achieve that, we proposed an approach using NLP technology and dictionary (or corpus) to find synonyms as alternative terms for original search terms. We constructed three sets to test our approach. The P@5 was improved by 0.767 on average, compared to the baseline method (without using our approach). It is helpful to accelerate and advance health-related computing and EHR sharing.

## Data Availability

The datasets used and analysed during the current study are available from the corresponding author on reasonable request.

## Abbreviations

EHR: Electronic health record
NLP: Nature language processing
HMC: Healthcare modelling collaboration
CS-GAN: Category sentence generative adversarial network
MLSREN: Multi-level semantic representation enhancement network
DB: Ndeep belief network
UMLS: Unified medical language system
SNOMED-CT: Systematized nomenclature of medicine clinical terms
AP: Average precision
P@3: Precision at 3
P@5: Precision at 5
MAP: Mean average precision
Low Level Set: Test set with low medical professional level
Medium Level Set: Test set with medium medical professional level
High Level Set: Test set with high medical professional level
